# Changes in Learning and Foraging Behaviour within Developing Bumble Bee (*Bombus terrestris*) Colonies

**DOI:** 10.1371/journal.pone.0090556

**Published:** 2014-03-05

**Authors:** Lisa J. Evans, Nigel E. Raine

**Affiliations:** School of Biological Sciences, Royal Holloway University of London, Egham, United Kingdom; Universidade de São Paulo, Faculdade de Filosofia Ciências e Letras de Ribeirão Preto, Brazil

## Abstract

Organisation in eusocial insect colonies emerges from the decisions and actions of its individual members. In turn, these decisions and actions are influenced by the individual's behaviour (or temperament). Although there is variation in the behaviour of individuals within a colony, we know surprisingly little about how (or indeed if) the types of behaviour present in a colony change over time. Here, for the first time, we assessed potential changes in the behavioural type of foragers during colony development. Using an ecologically relevant foraging task, we measured the decision speed and learning ability of bumble bees (*Bombus terrestris*) at different stages of colony development. We determined whether individuals that forage early in the colony life cycle (the queen and early emerging workers) behaved differently from workers that emerge and forage at the end of colony development. Whilst we found no overall change in the foraging behaviour of workers with colony development, there were strong differences in foraging behaviour between queens and their workers. Queens appeared to forage more cautiously than their workers and were also quicker to learn. These behaviours could allow queens to maximise their nectar collecting efficiency whilst avoiding predation. Because the foundress queen is crucial to the survival and success of a bumble bee colony, more efficient foraging behaviour in queens may have strong adaptive value.

## Introduction

Behavioural variation in social insects exists at multiple levels [1], and whilst much is known about variation in behaviour among species [2]–[4], colonies [5]–[10], genetic lines [11], [12] and castes [13]–[15], until recently little attention has been paid to the variation in behavioural types among individuals within a colony [16]–[20]. Like most animals, social insect individuals will alter their behaviour, for example their level of aggression, depending on the ecological situation. For instance, if a social insect colony is attacked by a predator, its members will behave aggressively in an attempt to protect their nest. However, some individuals are consistently more aggressive irrespective of the context or situation [21]. This concept forms the basis for behavioural syndromes [21], [22] and animal personality [23], whereby individuals exhibit different behavioural types/temperaments. The behavioural types of individuals are important in social insects as the decisions, actions, and fitness of a functioning colony are influenced by the behaviour of its members [10], [21], [24]–[26].

Previous studies looking at behavioural types among social insect individuals have only characterised behavioural differences among a small number of colony members over a short time period (typically less than three weeks [16]–[20]). As social insect colonies can persist for much longer periods (in some cases decades) and produce thousands of individuals, these studies provide only a ‘snapshot’ of the potential variation among individuals [27]. Therefore the possibility of directional changes in behavioural types at the individual (or colony) level has not yet been addressed, i.e. whether the first individuals to emerge behave differently to later emerging individuals in a colony. In theory such differences in behaviour over the course of colony development could be produced by variation in environmental conditions (e.g. temperature, odour, and food provisioning) during larval development and/or epi-genetic effects [28]. This is a fundamental question when studying colony development because the frequency of individual behavioural types at any point in time has the potential to affect overall colony behaviour and/or performance.

As colony demands change during development, we might expect the prominent behavioural type(s) to change in response. This could be especially true in colonies that begin with a single foundress (e.g. bumble bees, and some ant and termite species) that sequentially produces workers. Here, colony development coincides with extensive ecological and physiological changes, as well as an increase in the number of individuals present. For example, in many bumble bee species the foundress (queen) initiates a colony in early spring when weather conditions can be unfavourable and there are typically few floral resources [29]. This could mean the task of acquiring sufficient food is difficult for the foundress and the first workers, particularly because incipient (early stage) colonies have a high larva/worker ratio [30]. When the colony reaches maturity in summer, weather conditions tend to be more favourable and floral resources more abundant and diverse [31], [32].

The value of individuals will also vary depending on the developmental stage of the colony. During the founding stage, the foundress is essential as she is solely responsible for all aspects of brood care and foraging [33]. In an incipient colony, each individual is still highly valuable to colony success. If an individual dies, or is impaired [34], the colony may struggle to perform essential tasks (e.g. brood care or foraging). However, in a mature colony the value of each individual is lower because there is greater redundancy in the system: i.e. ‘resting’ individuals can be recruited to take-over a particular task. Even if the performance level of all individuals recruited to a task is comparatively poor, the task will still be completed [35]. Furthermore, in a mature colony a worker's effort is not lost if it dies because the colony has the capacity to maintain worker production [36].

One way social insects cope with such ecological, physiological and ergonomic changes is to produce workers that are morphologically distinct in their size and/or form [37]. There are many examples among social insects where a colony initially produces smaller workers, giving rise to larger workers as they develop, such as ants (*Solenopsis invicta* [38], *O*e*cophylla smaragdina* [39] and *Myrmica rubra* [40]), wasps (*Vespula vulgaris* [41]) and bumble bees (*Bombus terrestris* [42]). Whilst debate about the role of polymorphic workers continues, there is evidence that smaller workers are important for brood rearing [43], [44] and are more resistant to starvation [45]. This could suggest that smaller workers are better suited to the harsher conditions faced by an early stage colony.

While potential changes in the behavioural types present within a colony over time have yet to be explored, results from interspecific comparisons indicate predictable differences in the behavioural types present in different sized colonies [37], [46]. For example, ant species with larger colonies reportedly use foraging strategies such as group hunting, in which foragers exhibit a high tempo (activity level) and have a high encounter rate with prey [46]. In contrast, ant species with smaller colonies tend to forage as individuals and rely on stealth, rather than high tempo, to catch prey [35], [46].

To determine whether the behavioural types present within a eusocial insect colony do change over time, we used *Bombus terrestris* as a model system because they produce annual colonies of up to a few hundred workers, enabling us to assess the foraging behaviour of individuals across the entire period of colony development [47]. In addition, the role of individual foragers may be more important in bumble bees than other social insect species, as they largely explore the environment as individuals when looking for food [48], [49].

Using an ecologically relevant foraging task we assessed the decision speed and learning ability of nectar foragers throughout the developmental period of a colony, as these behaviours have important consequences for their foraging performance [24], [50]. Furthermore, because individual bees have a consistent propensity for both their decision making speed and learning performance [17], [18], [51], we were able to obtain comparable measures of foraging behaviour at different stages of colony development.

We hypothesised that the queen and the first workers to forage are of greater individual value to the colony, and would therefore (i) forage more cautiously to minimise risks of predation [52] and (ii) be faster learners due to greater pressure to find food under harsher foraging conditions. Despite being crucial for colony establishment, the behaviour of bumble bee queens outside the nest has received very little attention (but see [53]). Assessing the behaviour of both queens and their workers enabled us to compare behaviour between reproductive and non-reproductive castes, which has not previously been attempted.

## Methods

### Study Species

Seventeen *Bombus terrestris* queens, that had recently emerged from hibernation but had not yet started laying eggs, were obtained from Syngenta Bioline (Weert, the Netherlands). Each queen was placed inside a Perspex colony rearing box with a ball of pollen (sourced from Koppert Ltd, UK) and a transparent gravity feeder containing 50% (v/v) sucrose solution. Queens were kept in a dark, temperature controlled room and monitored daily. Eight (of 17) queens began to incubate an incipient pupal clump (typically this occurred within two weeks) and were each transferred to a separate wooden colony nest box and kept in the laboratory at ambient temperature (*ca.* 20°C). Some of these queens went on to produce workers which were subsequently assessed. High-frequency fluorescent lighting (>28 kHz) with Activa daylight tubes (Osram, Germany) was used in the laboratory to simulate natural daylight above the bee flicker fusion frequency [6]. Each wooden nest box was connected to a flight arena (120×100×35 cm) using a transparent Perspex tube. When foragers were not being pre-trained or trained sucrose solution was provided *ad libitum* from a gravity feeder in the flight arena. Pollen was provided *ad libitum* throughout the experiment from a petri dish in the nest box.

### Assessing Foraging Behaviour

#### Queen pre-training

During pre-training 20 bi-coloured blue and yellow artificial flowers were provided to give the colour naïve queens an equal opportunity to associate both colours as predictors of reward [6], [24]. The artificial flowers were squares (40×40 mm) constructed from equal areas of yellow (Perspex yellow 260) and blue (Perspex blue 727) Perspex. Each flower had a small well in its centre to hold sucrose solution. The flowers used for the queens were larger than those for workers (24×24 mm) because queens had difficulty landing on the smaller flowers. This difference in flower size is not expected to differentially affect measures of queen and worker decision making speed or learning ability, both because artificial flowers larger than 15 mm had no effect on worker search time at similar spatial scale [54], and the associative learning performance of bumble bee workers was equally good for large (38 mm) and small (29 mm) artificial flowers respectively [55]. The flowers were raised above the floor of the flight arena using colourless glass vials (40 mm high), and were arranged at random spatial positions within the flight arena.

We estimated the volume of sucrose collected by each queen in a single foraging bout to enable us to adjust the total volume of sucrose available (across all flowers) in the training phase (described below) to the crop capacity of the queen being trained. This was to encourage the queen to visit all the rewarding flowers before returning to the nest box. Estimations were calculated by multiplying the average number of flowers visited over three foraging bouts by 10 µl (the sucrose volume in each flower). An additional 10% was added to compensate for the queen not being able to find all the rewarding flowers.

#### Queen training – decision speed

Six out of eight queens were successfully pre-trained to forage on the bi-coloured flowers (36–48 h per queen) and were subsequently trained at approximately the same stage of colony development (when multiple pupal clumps were evident, approximately two weeks before the first workers emerged). Prior to training the nectar pot inside the nest was partially drained with a pipette (leaving a similar volume for all individuals), to motivate the queen to forage. During training, the arena contained 10 blue and 10 yellow flowers (both 40×40 mm). Each yellow flower was rewarded with a volume of 50% sucrose solution determined for each queen during the pre-training phase, while the blue flowers were empty (unrewarded). *Bombus terrestris* has an innate preference for blue [56], [57], so in the training phase we were interested to examine how long it took each bee to evaluate its options and make the decision to probe one of the rewarding (but innately less attractive) yellow flowers. A queen was regarded as choosing a flower when they approached (i.e. oriented towards a flower with their head <2 cm away) or landed on it. If the queen landed on a flower and fed (or attempted to feed) by extending its proboscis this was recorded as a probe.

Queen ‘decision making speed’ is defined here as a measure of ‘feeding latency’; the time elapsed between flight initiation and the first probe of a yellow (rewarding) flower. An example of a queen that made decisions rapidly would be one that readily approached and/or landed on the blue flowers and after finding they were unrewarding, visited a yellow flower. A queen that spent more time making decisions could spend a long period surveying the flowers from a distance, then approach a large number of flowers of both colours before landing on and probing a yellow flower. As the colours used in the training phase were both present in the bi-coloured (pre-training) flowers it is unlikely that choice behaviour observed will be influenced by neophilia or neophobia. In all cases it took multiple foraging bouts before the queen probed a yellow (rewarding) flower. The time spent in the arena in each of these bouts were summed to give a ‘decision making speed’ for each queen. Two of the six queens were excluded from the experiment as they spent over 30 minutes (across multiple bouts) in the arena without probing a yellow flower.

#### Queen training - learning performance

We continued to record the flower choice sequence of each queen after the first time it probed a yellow (rewarding) flower to assess the dynamics of associative colour learning. Choosing a yellow flower was considered ‘correct’ while choosing a blue flower was an ‘error’ [24]. The choice sequence made by each bee was recorded using Etholog software [58] from the first time it entered the arena in the training phase until it had made at least 99 choices after the first time it fed from a rewarding yellow flower (i.e. at least 100 flower choices in total). All flowers were replaced and their spatial position re-randomised between foraging bouts to prevent bees using scent marks or spatial cues as predictors of rewards. Four of the six pre-trained queens were successfully trained. Training lasted up to 12 h per queen because there were often long intervals between foraging bouts. Three of the trained queens, and one of the queens that did not successfully complete the training phase, continued to produce a colony with at least 100 workers.

#### Worker pre-training

Newly eclosed workers produced by three of the successfully trained queens, and a fourth queen that did not complete the training phase, were individually marked with numbered tags (Opalith tags; Christian Graze KG, Germany) on the day of emergence, so we knew the previous foraging experience and age of each individual when they were trained. Tagging also provided a measure of colony size (number of workers present). A Perspex tunnel with built in shutters replaced the Perspex tube between the flight arena and nest box. The shutters were used to control the flow of workers in and out of the flight arena, and enabled us to assess each bee individually. Foragers (n = 89) were pre-trained to forage on bi-coloured, blue and yellow, artificial flowers (24×24 mm). During the pre-training period all the bi-coloured flowers contained 5 µl of 50% sucrose solution. Bees that left the colony and fed on the bi-coloured flowers for at least five consecutive foraging bouts were selected for training.

#### Worker training – decision speed and learning

The training procedure for worker decision making speed and learning ability (up to 90 min per worker) were carried out as described above for queens. Workers that did not probe a yellow flower within 30 minutes were excluded from the experiment. In order to recruit new foragers, trained foragers were removed and frozen. Thorax width measurements were taken for each of the trained bees using digital callipers and recorded as a measure of worker body size. Training workers from each colony continued at least until worker production ceased and the first sexuals (males and gynes/new queens) began to emerge. To generate a record of the individuals foraging for nectar, each colony was observed for 10 minutes at least twice per day. A bee was considered a forager if it was observed on the sucrose feeder on at least two separate observations.

### Analysis

**Figure 1:**
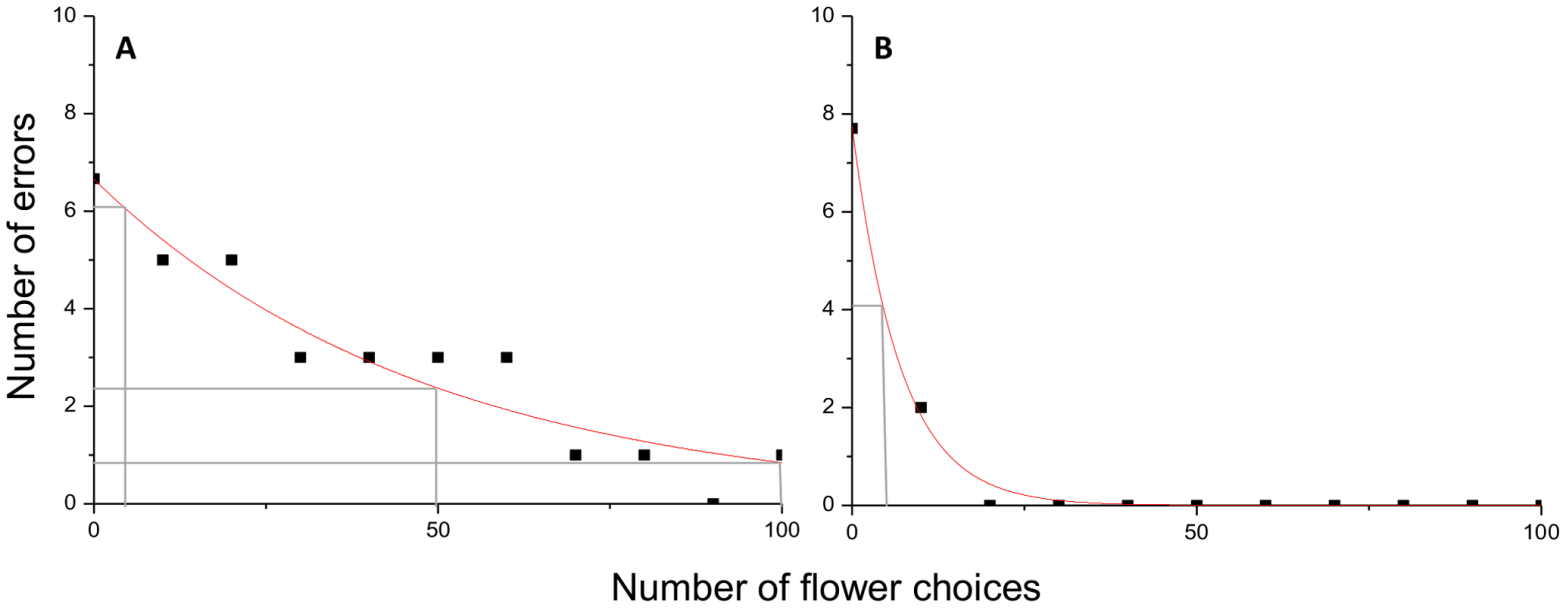
Example learning curves for a slow and a fast learning bee. These curves illustrate how the performance of two bumble bee (*B. terrestris*) individuals change during the learning task. Black squares indicate the number of errors made by each bee in groups of 10 flower choices. Red curves, fitted to the empirical data using the formula: y = y0+Ae^−x/t^ [24], illustrate how the performance of each bee changes during the task. Grey lines indicate the number of errors made by bees after 5, 50, and 100 flower choices. These three values were summed to generate a single measure of learning performance (Learning Performance Index: LPI) that accounted for the rate of change in performance (slope of the learning curve: t), the shape of the learning curve, and the saturation performance level (y0). Bee (A) is an example of a slower learner and has a higher LPI = 9.4, while bee (B) is an example of a fast learner and has a low LPI = 4.1.

Learning curves; first-order exponential decay functions (y = y0+Ae^−x/t^), were fitted to the flower choice data for each bee using Microcal Origin pro 8.6. In this equation ‘x’ is the number of flower choices the bee made after its first yellow flower probe, and ‘y’ is the number of errors. ‘y0’ is the saturation performance level - the number of errors made by the bee when they reach a performance plateau. ‘t’ is the decay constant of the curve - a measure of learning speed (rate of change in task performance) and ‘A’ is the curve amplitude. The starting point for each bee's learning curve was the proportion of errors made (number of blue flowers chosen) before a bee probed a rewarding yellow flower for the first time. Flower choices made by each bee after and including the first time it probed a rewarding yellow flower were evaluated as number of errors (blue flower choices) in each group of 10 choices. The learning curve was fitted to these 11 data points, i.e. start point and subsequent 10 groups of 10 flower choices, for each individual bee [24] (Figure 1). To generate a single Learning Performance Index (LPI) that took into account the rate of change in performance (slope of the learning curve), the shape of the learning curve and variation in the saturation performance level, we summed the number of errors made by each bee when it had made 5, 50, and 100 choices after probing a rewarding flower for the first time. This produced a learning score out of 30. Low LPI values are indicative of faster learning while high values indicate slower learners (Figure 1).

The foraging behaviour of queens was compared to that of their workers using a one-way ANOVA. As many workers were being compared with a single queen, bootstrapping (set to 1000 iterations) was used to generate robust 95% confidence intervals (using SPSS v.20). Spearman's rank correlations were calculated for colony age and foraging behaviour (decision making speed and learning ability) for the trained workers in each colony. A General Linear Mixed Effects Model (GLMM), including ‘colony’ as a random factor, was used to determine whether there was a relationship between colony age and foraging behaviour across colonies. Worker ‘age’ (days since eclosion) and ‘size’ (thorax width) were included in the model as fixed covariates. Worker age and size were included in the model because previous research has shown correlations between these variables and learning ability in some colonies [6], [59]. The data for ‘colony age’, ‘worker age’, and ‘decision speed’ were log_10_ transformed to normalise residuals. All data analyses were performed in SPSS (v.19) unless otherwise stated.

## Results

### Queen Worker comparison

Each queen took between four and seven foraging bouts (over a period of 7–12 hours) to probe a rewarding yellow flower. The percentage of blue flowers queens chose before probing a rewarding yellow flower (an indication of innate blue preference) was not significantly different from that of their workers (Table 1). In two colonies there was no significant difference in the total number of flower choices made by queens and workers prior to probing a rewarding flower (one-way ANOVA, p-value generated in pairwise comparison with bootstrapping: colony 2, *F*_(1,16)_ = 1.906, *P* = 0.19; colony 4, *F*_(1,17)_ = 0.830, *P* = 0.07, whilst in colony 1 the queen made significantly more choices (all approaches) than her workers (one-way ANOVA, p-value generated in pairwise comparison with bootstrapping: *F*_(1,22)_ = 4.770, *P* = 0.001: Table 1).

**Table 1:** A comparison of the number of flower choices, decision speed, and learning performance of queens and workers.

	% of initial blue flower choices		Total number of choices		Decision speed (sec)		LPI	
Colony	Queen	Worker	Queen	Worker	Queen	Worker	Queen	Worker
1	87	65 (±3.0)	68	26 (±4.3)	635.5	139.8 (±32.1)	†0	6.8 (±0.72)
2	80	80 (±11.5)	109	87 (±12.7)	1963	568.1 (±90.1)	5.24	7 (±0.91)
3	-	76 (±10.7)	-	55 (±13.3)	-	320.9 (±95.6)	-	7.6 (±0.57)
4	81	88 (±9.5)	71	37 (±9.5)	909.1	196.6 (±52.8)	1.29	7.2 (±0.71)
*5	86	-	96	-	1216.1	-	1.34	-

* The queen in colony 5 produced only 6 workers and has therefore been excluded from our analysis.

† Queen made no errors so has LPI = zero.

Data shown are the percentage of blue flower choices made by queens and workers from each colony prior to making their first probe on a yellow flower, the total number of flower choices made, the time taken to feed from a (yellow) rewarding flower (decision speed), and the Learning Performance Index (LPI). Worker data are presented as colony mean (± SE) values.

**Figure 2:**
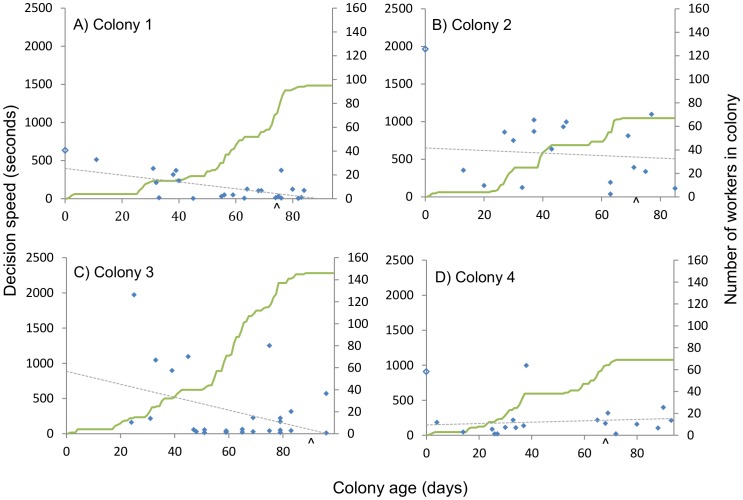
The decision speed of individual foragers as a function of colony age. Data shown indicate the decision speed of each individual trained in colonies 1(A), 2(B), 3(C), and 4(D) plotted against the number of days after the emergence of the first worker (colony age). Open symbols represent queens and filled symbols are workers. The curved green lines indicate colony growth (measured by the number of marked workers present in the colony: see y-axis on right hand side) and dashed lines show non-significant correlations (excluding the queen) between decision speed and colony age. A caret on the x-axis indicates the day the first sexuals (males) eclosed.

**Figure 3:**
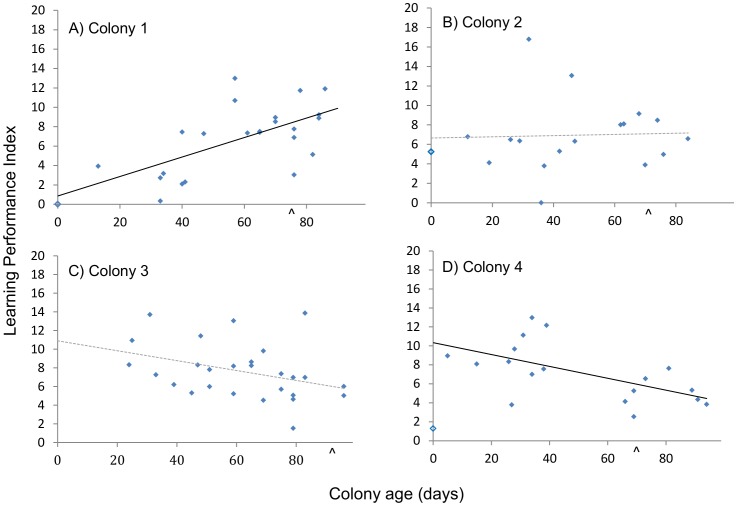
The learning performance of individual foragers as a function of colony age. Data shown indicate the learning performance (Learning Performance Index) of each individual trained in colonies 1(A), 2(B), 3(C), and 4(D) plotted against the number of days after the emergence of the first worker (colony age). Open symbols represent queens and filled symbols are workers. Straight lines are the correlation (excluding the queen) between learning performance and colony age. Significant correlations are represented by solid lines and non-significant correlations with dashed lines. A caret on the x-axis indicates the day the first sexuals (males) eclosed.

We found consistent differences between queens and their workers in both decision speed and learning performance. All queens trained spent significantly more time in the flight arena than workers before probing a rewarding flower for the first time (one-way ANOVA, p-value generated in pairwise comparison with bootstrapping: colony 1, *F*_(1,22)_ = 2.041, *P* = 0.002; colony 2, *F*_(1,16)_ = 5.814 *P* = 0.002; colony 4, *F*_(1,17)_ = 0.514, *P* = 0.003: Table 1 and Figure 2), meaning that queen decision speed was slower than that of workers from their colony. Two of the three queens performed significantly better than their workers in the learning task (one-way ANOVA, p-value generated in pairwise comparison of LPI with bootstrapping: colony 1, *F*_(1,22)_ = 1.903, *P* = 0.002; colony 4, *F*_(1,17)_ = 1.947, *P* = 0.002) indicating that they made fewer errors. While there was no significant difference between the learning ability of the queen and workers in colony 2 (one-way ANOVA, p-value generated in pairwise comparison of LPI with bootstrapping: *F*_(1,16)_ = 0.873, *P* = 0.182) the queen notably made fewer errors than 71% of her workers (Figure 3).

### Worker foraging performance

#### Across colonies

The foraging performance of 85 workers (17–27 per colony) was successfully assessed, representing 47% of all foragers produced from four colonies (Table 2). Workers were trained throughout colony development, from four to 96 days following the first worker eclosion.

**Table 2:** The number of foragers trained at the different stages of colony growth/development.

Colony	Nectar foragers trained						
	Queen trained	Colony age (days)				Total n. (% foragers)	
		0–20	21–40	41–60	61+		
1	Y	1	5	4	13	**23**	**(45)**
2	Y	2	5	3	7	**17**	**(53)**
3	N	0	5	8	14	**27**	**(47)**
4	Y	2	8	0	8	**18**	**(44)**

Data shown indicate whether the queen was successfully trained in each colony (Yes/No), the number of nectar foragers trained per colony at different stages of colony development (time periods are 0–20, 21–40, 41–60 and 61+ days after the emergence of the first worker: colony age), and the total number of workers (and percentage of the colony nectar foraging force) trained.

Overall, we found the decision speed of workers was not affected by colony age (GLMM *F*_(1, 70.35)_ = 0.215, *P* = 0.644), worker age (GLMM *F*_(1, 72.81)_ = 0.013, *P* = 0.910), or worker size (thorax width: GLMM *F*_(1, 71.91)_ = 0.169, *P* = 0.683). Likewise, learning performance (LPI) was also not affected by colony age (GLMM *F*_(1, 73)_ = 0.292, *P* = 0.591), worker age (GLMM *F*_(1, 73)_ = 0, *P* = 0.999), or worker size (thorax width: GLMM *F*_(1, 73)_ = 0.403, *P* = 0.528).

#### Within individual colonies

There was no significant change in worker decision speed with colony age in any of the four colonies (Figure 2). However, learning performance did change with colony age in two out of the four colonies, but in different ways. Worker learning performance indices (LPIs) increased significantly, indicating a trend towards poorer learning performance, with colony age in Colony 1 (Spearman's ρ = 0.584, *N* = 23, *P* = 0.003) and decreased significantly, indicating a trend towards better learning performance, with colony age in Colony 4 (Spearman's ρ = −0.509, *N* = 18, *P* = 0.031). However, there was no significant relationship between learning performance and colony age in Colonies 2 and 3 (Figure 3).

We found a significant positive relationship between worker age and colony age in Colonies 2 and 4 (Spearman's ρ = 0.698, *N* = 17, *P* = 0.002; ρ = 0.737, *N* = 18, *P*<0.01 respectively), indicating a tendency for older workers to forage when these colonies were more developed (contained more workers). However, there was no correlation between worker age and colony age in Colonies 1 and 3, or between worker size and colony age in any of the four colonies.

## Discussion

Here we assessed whether the decision speed and/or learning ability of nectar foragers would change over the course of colony development. The foundress queens (each colony's first forager) showed consistent differences in foraging behaviour compared to their workers: queens took between 3.5–4.6 times longer than workers to decide to visit a yellow (rewarding) flower. That is, they spent more time inspecting their foraging options from afar (and in one case approached more flowers) before visiting a rewarding, but innately less attractive, yellow flower. It is unlikely that their slow decision speed corresponds to a stronger innate preference for blue, as the number of blue flowers approached before visiting a yellow flower was not greater than their workers. Their slower decision speed may be indicative that a foundress queen makes more cautious foraging decisions. This makes evolutionary sense, as the death of a bumble bee queen would mean her colony would perish long before producing reproductive offspring. This finding is in line with the predictions of the ‘CD Model’ [60], [61] that solitary bees should adopt less risky foraging strategies than social bees because if they die prematurely there are no workers to continue rearing their brood. In bumble bees the foundress queen is essentially behaving like a solitary female, acting as the sole provider of food while rearing her first batch of workers. Thus she may also forage cautiously in order to minimize the chance of potentially hazardous errors.

While the potential adaptive value of this behavioural trait for queens seems clear, the extent of the difference between the decision speed of workers and queens is somewhat surprising; as evidence from other taxa indicates that individuals with larger body size experience reduced predation risk [62] due to them having fewer predators. This may be the case for bumble bee queens as they are likely to be too large for crab spiders, a major bee predator [63]. However, whilst forager body size has been assumed to affect vulnerability to capture by predators, variation in body size among *Bombus impatiens* workers does not reliably predict their response to an attack from a crab spider [64]. Jones & Dornhaus [64] suggested this could be due to trade-offs associated with increased body size: for instance, larger body mass can reduce flight performance which may lead to increased vulnerability to capture [65]. Large-bodied individuals can also be less agile [66], [67], so larger foragers may be less able to escape from a predator once they have landed on a flower.

Furthermore, there are other predators to which bumble bee queens are certainly susceptible whilst visiting flowers, including conopid flies (that wait on flowers for foraging bees which they then parasitize)[68] and birds [47], [69]. In some situations larger, more noticeable queens could be more vulnerable to predation, which may lead them to display more risk averse behaviour [70].

There could be other problems associated with flying around without landing on a flower: for instance, increased energy usage and wing wear. As larger-bodied individuals/species tend to have a lower cost of flight relative to smaller individuals [71], large queens can afford to spend more time surveying a new flower patch before committing to visiting a less attractive (potentially risky) flower type than smaller workers. Furthermore, workers will have a higher relative energy requirement for flight due to their smaller body size [72] meaning it may not be possible for them to spend as much time as a queen surveying forage options before deciding which flower(s) to visit. Evidence to date also suggests that wing wear is not related to duration of flight activity [73]. Overall, this suggests that the longer decision times of queens are unlikely to be limited by differences in energetic or physical constraints of flight compared to workers.

We also found a trend for queens to be faster than their workers at learning to associate yellow as a predictor of reward. As queens are appreciably larger than workers we would expect their visual acuity to be greater [74], which might be one explanation for this difference in learning performance. However, the ability to detect an object is also determined by its shape, size and colour [75]–[77]. In the current study, the flowers used for both the queen and worker pre-training and training were both relatively large compared to both bee body size and the flight arena dimensions. As a result, all flowers would have been easily detected by bees within the flight arena and the two colours used were easy for them discriminate [6], [51]. It is therefore unlikely that observed differences in queen performance were a result of either their superior visual acuity or discrimination performance.

Queens were exposed to a greater area of yellow during the pre-training phase which could have led them to form a stronger association between yellow and reward prior to being trained. However, if this was true then we would have expected queens to have either a quicker ‘decision speed’ and/or make fewer choices before probing a yellow flower in the training phase and this was not the case. The ability of foundress queens to quickly form an association between a colour and reward is perhaps more likely to indicate that they are in fact excellent learners.

The founding stage of a bumble bee colony, when the queen is solely responsible for foraging and brood care, is the most critical period in their life cycle [33], [47]. In other species of eusocial insect high mortality in independently founded colonies is thought to have been a strong selection pressure for the evolution of cooperation between unrelated co-foundresses [78], [79]. Perhaps in bumble bees the ability of the queen to learn quickly helps to increase the colony's chance of survival. For instance, learning quickly which flowers to visit, and those to avoid, may enable queens to maximise their nectar collecting efficiency and allow them to return promptly to their brood, which needs to be maintained ca. 30–32°C [68]. Extended and/or unsuccessful foraging trips are likely to cause brood temperatures to drop. Possible consequences of this include a delay in brood emergence [80], a reduction in the quality of brood reaching maturity [81], [82] and increased brood mortality. All of which are likely to result in lower colony survivorship and/or reproductive success.

This study provides an insight into the learning and foraging performance of mated foundress queens and to our knowledge it is the first experimental study that specifically investigates either the learning or foraging behaviour of bumble bee queens (Clark & Dukas used gynes rather than workers to look at bumble bee performance in a categorization tasks [61]). Given the importance of a bumble bee queen not only for the success of their colony but also its existence, this is an area that should be explored much more extensively.

Contrary to our expectations, there was no predictable change in the foraging behavioural types of workers with colony development. When we looked at the colonies individually there was evidence that learning ability (but not decision speed) changed with colony development in two out of the four colonies. However, the way in which these foraging behaviours changed was not consistent across colonies. In one colony learning ability improved with colony development while in another colony it decreased. This result is similar to inconsistent trends among bumble bee colonies shown for both worker age and body size in relation to learning performance [6]. Raine and colleagues concluded that whilst there was no clear effect of age on learning performance across the 16 tested colonies, some colonies may have a genetic predisposition for age to affect their learning performance as seen in honey bees [83], [84]. In Colonies 2 and 4 we found a positive relationship between colony age and worker age, suggesting nectar foragers did not leave the colony until they were older when these colonies were more mature. If Colony 4 (but not Colony 2) had a genetic predisposition for worker age to affect their learning performance then this interaction could explain the observed correlation between colony age and learning performance in this colony. However this would not explain the significant relationships observed between colony age and learning performance in both Colonies 1 and 4. It would be interesting to see whether similar patterns were still observed if appreciably larger numbers of colonies were assessed using this time intensive protocol.

Intracolony variability in behavioural types could benefit colony survival because it enables more effective response to environmental variation [19], [50], [85]. For example bees exhibiting different foraging strategies can be more or less efficient at collecting nectar depending upon the relative abundance of rewarding flowers present [19]. When foraging in a patch containing two similarly coloured artificial flowers, one colour containing sucrose solution the other unrewarding, slow-accurate foragers were more efficient when few of the flowers were rewarding, whereas fast-inaccurate bees were more efficient when many of the flowers contained rewards [19]. If variation in forager behavioural types (e.g. in learning ability) helps the colony to deal with environmental variation, it is reasonable to assume that this benefit will accrue as soon as the colony is large enough to produce multiple foragers. This might explain why we observed no consistent directional changes in behavioural type variation over the course of colony development.

Whilst some studies have provided ‘snapshots’ of behavioural variation that exist within a colony, we examined here for the first time how changes in the behavioural composition of a colony's foragers might change over its developmental period. While there was no predictable change in foraging behaviour of *B. terrestris* workers depending on the stage of colony development when they began to forage, we show that behavioural phenotypes present can change in individual colonies over time. In light of these results the possibility of directional change being associated with colony developmental stage should be considered in future studies describing behavioural traits at the individual or colony level. In addition, we showed that the foraging behaviour of foundress queens was predictably different from that of their workers. This raises some interesting questions regarding the evolutionary importance of queen foraging behaviour and how this affects colony survivorship, growth and reproductive output.
